# Preliminary Phytoconstituent Analysis and In Vitro Evaluation of Antibacterial and Antioxidant Activities of *Verbascum sinaiticum* Benth Leaf Extracts

**DOI:** 10.1155/jotm/6626328

**Published:** 2026-06-18

**Authors:** Ayalew Temesgen Wodajo, Birhan Amanie Mekonnen, Shewaye Lakew Mekuria, Gizachew Mulugeta Manahelohe

**Affiliations:** ^1^ Department of Chemistry, College of Natural and Computational Sciences, University of Gondar, Gondar, 196, Ethiopia, uog.edu.et

**Keywords:** antimicrobial assay, antioxidant activity, DPPH assay, flavonoids, FRAP assay, phenols, *V. sinaiticum*

## Abstract

*Verbascum sinaiticum* Benth. commonly known as “Qetetina” in Ethiopia, the family Scrophulariaceae has considerable importance in traditional medicine worldwide because of its antioxidant and anti‐inflammatory activities. *V. sinaiticum* is one of the commonly used traditional medicinal plants in Ethiopia for treating wounds, cancer, and animal trypanosomiasis. Hence, the present study was undertaken to screen the phytochemical constituents and evaluate the antibacterial and antioxidant activity of *V. sinaiticum* chloroform and methanol leaf extracts against two Gram‐positive bacteria (*Staphylococcus aureus* and *Streptococcus pneumoniae*) and two Gram‐negative bacterial strains (*Klebsiella pneumoniae* and *Escherichia coli*) by the agar disc well diffusion method. The study revealed the presence of phenols, flavonoids, steroids, saponins, terpenoids, alkaloids, and tannins. Thus, the present investigation was undertaken to estimate total phenols and flavonoids in chloroform and methanol extracts of *V. sinaiticum* leaves. In vitro antioxidant activity was also evaluated in the methanol and chloroform extracts using DPPH and FRAP assays. The DPPH assay showed that standard IC_50_ for ascorbic acid (AA) was 23.79 μg/mL, whereas for methanol extract the IC_50_ value was 29.88 μg/mL and 86.38 μg/mL for chloroform extract, while the FRAP assay showed standard IC_50_ value for AA as 46.2 μg/mL, 80.3 µg/mL for methanol extract and 201.2 µg/mL for chloroform extract with *p* values < 0.05, which indicates that methanol extract exhibits higher antioxidant activity than chloroform crude extract. The methanolic extracts of *V. sinaiticum* leaves showed moderate amounts of total phenolic and flavonoid contents, 7.81 ± 0.14 mg GAE/g (milligrams of gallic acid equivalent) and 14.20 ± 0.98 mg QE/g of dried crude, respectively. Using the agar well diffusion method, the two extracts were evaluated against Gram‐positive (*S. aureus* and *S. pneumonia*) and Gram‐negative (*Salmonella typhi* and *E. coli*) bacterial strains. The leaf extract displayed a greater impact on both Gram‐positive bacterial strains and Gram‐negative bacterial strains. 21.7 ± 0.6 against *E. coli* at concentrations of 100 and 50 μg/mL and 19.7 ± 0.6 against *S. typhi* compared to positive control ciprofloxacin 23.6 ± 0.4 and 28.6 ± 0.6, respectively. The minimum inhibitory concentration assay was determined using the concentrations of 100 mg/mL, 50 mg/mL, 25 mg/mL, and 12.5 mg/mL by the agar well diffusion method. All extracts had shown a significant antibacterial activity (*p* value < 0.05) within the average inhibition zone ranging from 10.2 ± 1.3 to 21.7 ± 0.6. *V. sinaiticum* leaves showed strong antibacterial activities against *S. aureus*, *S. pneumonia*, *E. coli*, and *S. typhi*. Therefore, it is evident from this study that the highest therapeutic efficacy possessing majority of secondary metabolite classes of compounds in the leaf extract of *V. sinaiticum*, which can be quantified for application in the pharmaceutical industry. Especially, flavonoids and tannins were strongly detected. Finally, the authors recommend future isolation and structural characterization of specific bioactive compounds, as well as in vivo toxicity and biological studies.

## 1. Introduction

Ethiopians have long been familiar with traditional medicine. Most of the knowledge is oral and has been passed down from generation to generation by knowledgeable elders, practitioners of healing, and regular people [1]. Because traditional healers and local pharmacopeias are accepted in Ethiopian culture, traditional medicine is used by up to 80% of the population [2]. Plants are essential to human requirements daily. Typically, they are utilized as food, cosmetics, flavors, medications. and decorations. Historically, plants have been utilized in medicine to cure a wide range of illnesses in humans, including infectious diseases brought on by various microbes. Many plant extracts have found widespread application in medicine, particularly in the treatment of infectious disorders. Ethiopian herbs have demonstrated highly promising therapeutic potential for treating a range of microbiological illnesses [3].

*Verbascum sinaiticum* (Scrophulariaceae) is a biennial herbaceous species that is native to Ethiopia. It is also known by other colloquial names such as “Qetetina, yeferes zeng or Yahiya joro” in Amharic, “Gurra Harre,” and “Abokena” in Afan Oromo, and “Tirnake,” “Handega,” or “Kunama Luta” in Tigrigna. With over 360 species, *Verbascum* is the largest genus in the figwort family, Scrophulariaceae. Most of the species are found in tropical highland regions with moderate temperatures [4].

This popular plant is found in all parts of Ethiopia, Sudan, Somalia, Kenya, Israel, Jordan, Syria, Afghanistan, and the former southern region of Russia. It grows widely throughout Africa and the Mediterranean region. It was also imported as a weed to Yemen [3, 5]. The order Lamiales includes the Scrophulariaceae, which have about 5850 species and roughly 306 genera. It is a perpetually flowering herb that grows to a height of 50–160 cm. Its thick mat of soft white hair covers the leaves and stems, making it prominent near roadways. Many iridoids have been isolated from Verbascum. Among them was aucubin, which was first isolated from *Aucuba japonica* Thumb and exhibited marked liver‐protective activity against CCl_4_ [6].

Phytochemical analysis of *V. sinaiticum leaves* has revealed the presence of flavonoids and phenolic compounds, including verbascoside, apigenin‐7‐glucoside, arenariosides, cistanosides, caffeic acid, chlorogenic acid, quercetin, myricetin, and kaempferol [7]. Additionally, flavonolignans and flavones have been isolated from the aerial parts of *V. sinaiticum*.

The concentration of phenolic acid, iridoid, phenylethanoid glycoside, and flavonoids in *V. sinaiticum* leaves may account for some of its beneficial effects on health [8, 9]. Numerous investigations on this plant have demonstrated its antimitotic, antibacterial, and antioxidant qualities [10]. In Ethiopia, powdered leaves of *V. sinaiticum* mixed with water are given orally, and the filtrate is added to the left ear and nose of animals for the treatment of animal trypanosomiasis, wound healing, stomach viral infection, cancer, sunstroke fever, abdominal diseases, diarrhea, colds, snake bites, hemorrhage, anthrax, tapeworm, abdominal dropsy, rheumatic pain, mental illness, amnesia, syphilis, gonorrhea, relapsing fever, chest diseases, elephantiasis, hepatitis, and as a hepatoprotective agent [11].

Notwithstanding the extant literature on this plant, additional research is still necessary to fully understand its antibacterial, antioxidant, and phytochemical characteristics [12, 13]. The impact of microbial infections is particularly noticeable in impoverished nations like Ethiopia, where access to contemporary medications is limited. Various in vitro studies on *V. sinaiticum* have demonstrated its notable antimicrobial activity and the presence of diverse chemical constituents. Given its traditional use in folk medicine for wound healing, combined with its in vitro antibacterial properties and secondary metabolites, it is both reasonable and necessary to conduct an in vivo evaluation of its wound healing potential. Such studies will provide a scientific basis for its traditional use as a healing agent [14].

Additionally, there is a critical need for scientific validation, standardization, and safety assessment to protect consumers from the risks of overdose or subtherapeutic dosing. The findings from this study could serve as foundational data for future research aimed at the identification, isolation, purification, and antioxidant activity determination of the active compounds. Ultimately, this could lead to the synthesis of lead compounds with enhanced wound‐healing properties. The present work intends to do phytochemical screening and evaluation of the total phenolic (TPC) and total flavonoid content (TFC) and possible antioxidation and antibacterial activity of *V. sinaiticum* leaves using different solvent extracts.

## 2. Experimental Part

### 2.1. Collection of Plant Materials

The leaves of *V. sinaiticum* were collected on November 24, 2024, from the Amhara Regional State, South Gondar Zone, Lay‐Gayint Woreda, at the specific site of Sali, Ethiopia. Then, they were thoroughly washed with water to remove dust and dried under shade at room temperature for 20 days. The dried leaves were ground using a kitchen blender to obtain the coarse powder and kept in an airtight container till further use. The plant species was authenticated by Mr. Abiyu Enyew, a botanist at the University of Gondar, College of Natural and Computational Science, Department of Biology, and AE 98 was given as a voucher number.

### 2.2. List of Chemicals and Reagents

#### 2.2.1. Chemicals and Reagents

All the chemicals and reagents used in the present study were of analytical grade. The chemicals and reagents included methanol (Merck, Darmstadt, Germany); anhydrous aluminum chloride and sodium nitrite (Fluka, Switzerland); L‐ascorbic acid 98% (vitamin C; BDH Chemicals Ltd., Pool, England); Folin–Ciocalteu reagent, gallic acid, sodium hydroxide, and anhydrous sodium carbonate, which were purchased from Loba Chemie (Mumbai, India); whereas 2,2‐diphenyl‐1‐picrylhydrazyl (DPPH) was purchased from Sigma‐Aldrich (Poole, Dorset, England, UK).

#### 2.2.2. Apparatus and Equipment

An electrical grinder (IAK–WERKE, Germany), electronic balance (Bosch, Germany), Whatman No. 1 filter paper (100 mm), rotary evaporator (Bibby RE200B, Sterilin Ltd., UK), and UV/Vis spectrophotometer (Sanyo SP75, UK) were used in the study.

#### 2.2.3. Extract Preparation

Phytochemical extraction is crucial, with solvent choice affecting the yield and concentration of bioactive compounds. Water, being more polar, is less versatile than methanol, which can extract a wider variety of phytochemicals, including some nonpolar compounds. Methanol consistently yields more classes of compounds, such as phenols and steroids, often in higher concentrations than water, which mainly extracts polar constituents like tannins. Consequently, methanol extracts typically show greater biological activity in assays due to their broader range of active ingredients [15, 16]. Furthermore, polar solvents recover higher TPC and TFC than nonpolar solvents, with methanol being more effective than hydromethanolic and water extracts due to its ability to dissolve more nonphenolic compounds [17]. Based on the effects of the extraction solvents on the phytochemical content, antioxidants, and antimicrobial properties of the plant extracts, the 80% methanolic extract had the highest TPC. In antioxidant tests, 80% methanol extract showed the strongest activity (lowest IC_50_ in the DPPH test). Antibacterial activity tests showed 80% methanol and cold‐water extracts were effective against *Escherichia coli* and *Staphylococcus aureus* (inhibitory zones 25 mm) [18].

Two hundred grams of powdered *V. sinaiticum* leaves were macerated with 2.5 L of chloroform for 72 h with occasional shaking (4 times per day), filtered through Whatman No. 1 filter paper (100 × 100), and the filtrate was evaporated by rotary evaporator (England; RE200B) to give 2.8 g crude extract. The dried (at room temperature) residue, after chloroform extraction, was then extracted with 2 L methanol (80%) for 72 h at room temperature with periodic shaking. 80% methanol was preferred to improve extraction efficiency by targeting a broader range of compounds due to its increased polarity and ability to interact with different cellular structures compared to pure methanol [19]. The methanol extract was filtered through Whatman No. 1 filter paper and evaporated to give 32.7 g crude extract. Each crude extract was then stored in a refrigerator for further investigation. The percentage yield of each crude extract was then calculated by using the following formula [20].

(1) Percentage yield%=Mass of crude extractsMass of dry sample×100.

### 2.3. Qualitative Phytochemical Analysis

To identify the active secondary phytochemical metabolites, a chemical analysis was conducted on *V. sinaiticum* crude leaf extracts of methanol and chloroform, following recognized protocols. The availability of secondary metabolites, including flavonoids, terpenoids, saponins, alkaloids, tannins, steroids, and phenolic compounds, was assessed [21].

#### 2.3.1. Test for Flavonoids

The results of Shendo’s test show the presence of flavonoids when 0.5 g of the two crude plant extracts are combined with 3 mL of ethanol, a piece of magnesium ribbon, and 3 mL of strong HCl. A pink hue occurs after a few minutes [22].

#### 2.3.2. Test for Steroids (Salkowski Test)

A volume of 2 mL of chloroform was added to acetic anhydride, then after concentrated H2SO4 was added carefully along the side of the test tube to the crude extract. The presence of steroids was identified by the blue‐green color formed in the lower chloroform layer [23].

#### 2.3.3. Test for Saponins (Foam Test)

After adding 2 mL of distilled water and shaking well, 0.5 g of each of the crude extracts of methanol and chloroform were added. The creation of a 1 cm layer of foam shows the presence of saponins [24].

#### 2.3.4. Test for Phenols (Ferric Chloride Test)

The presence of phenolic compounds is shown by the blush green or black coloration that results from treating 0.5 g of crude extracts with a few drops of 2% FeCl_3_ [25].

#### 2.3.5. Test for Terpenoids (Salkowski Test)

In a test tube containing two 0.5 g plant extracts, 2 mL of chloroform was added. The presence of terpenoids was next confirmed by adding strong sulfuric acid to the mixture, which produced a reddish‐brown interface [26].

#### 2.3.6. Test for Alkaloids

Each 2 mL extract used in the Wagner’s reagent test was dissolved in diluted hydrochloric acid before being filtered. Following that, Wagner’s reagent (iodine in potassium iodide) was applied to the filtrates. Alkaloids are present when a brown or reddish brown precipitate forms [27].

#### 2.3.7. Test for Tannin

A few drops of 10% lead acetate were added to 3 mL of each extract. The presence of tannins is indicated by the development of white precipitate [28].

### 2.4. Quantitative Phytochemical Determination

#### 2.4.1. Determination of TPC

A slightly modified technique of the Folin–Ciocalteu method was used to analyze TPC of chloroform and methanol *V. sinaiticum* extracts quantitatively [29, 30]. Gallic acid was used as a reference standard for plotting the calibration curve.

Concentrations of 2.5, 5, 10, 25, 50, 100, and 150 ppm of gallic acid were prepared in methanol. Concentrations of 0.1 and 1 mg/mL of plant extract were also prepared in methanol, and 0.5 mL of extracts were introduced into test tubes and mixed with 2.5 mL of Folin–Ciocalteu reagent and 2 mL of 7.5% sodium carbonate (detailed methods for preparation of standards are available in supporting information). After incubation for 90 min at room temperature, the absorbance against the prepared reagent blank was measured at 765 nm using a UV spectrophotometer. The concentration of total phenolic compounds in the extract was expressed as milligrams of gallic acid equivalent (GAE) per gram (mg GAE/g) of sample. All the samples were analyzed in triplicate [31].

#### 2.4.2. Determination of TFC

The TFC was determined using a modified aluminum chloride assay [24, 32]. Briefly, 0.5 mL methanol solution of each crude extract was added into the separate test tube; 1 mL quercetin standard solution was mixed with 2 mL of distilled water and 0.15 mL of 5% NaNO_2_ solution, followed by 5‐min incubation. Then, 0.15 mL of 10% AlCl_3_ was added, and the mixture was left for another 5 min. Following this, 1 mL of 1 M NaOH and 1.2 mL of distilled water were added, resulting in an orange‐yellow color. The analysis of total flavonoids was conducted by using a UV–vis spectrophotometer at a wavelength of 510 nm [33] with methanol serving as the blank. Quercetin was used as the standard, and all experiments were performed in triplicate. Flavonoid content was expressed as quercetin equivalents (mg QE/g) using a linear equation derived from the standard calibration curve [32, 34].

### 2.5. DPPH‐Radical Scavenging Activity

With a few minor adjustments, in accordance with the protocol [35], in brief, in a volumetric flask, 39 mg of DPPH were dissolved in 80% methanol to prepare 0.1 mM, with a final volume of 1 mL. Thus, prepared, purple‐colored DPPH free radical solutions were stored at room temperature for further use. 10 mg of crude extract of chloroform was dissolved in 10 mL of 80% methanol and then serially diluted to get 10, 25, 50, 100, 150, and 200 ppm, in a similar manner for methanol (10, 25, 50, 100, 150, and 200 ppm).

#### 2.5.1. Preparation of Control Solutions

Plant extracts are often dissolved in methanol because methanol efficiently extracts a wide range of phytochemicals, including phenolic compounds and flavonoids, which possess antioxidant properties. Ascorbic acid (AA) (vitamin C) is a polar compound, and it is dissolved in water because it is most soluble in water, a highly polar solvent, making water the ideal medium for preparing its standard solution for analysis [36].

A volume of 1 mL of 80% methanol was mixed with 2.5 mL of DPPH aqueous solution and prepared to compare the color change of the sample‐containing solutions to this control, which helps accurately calculate the scavenging activity of the antioxidant, ensuring the observed color reduction is due to the antioxidant rather than other factors.

Ten mg of AA standard was added into 100 mL of flask, dissolved in 10 mL of distilled water, and then distilled water was added up to the mark to prepare a 1000 ppm master stock solution. Subsequently, working solutions of 10, 25, 50, 100, 150, and 200 ppm were diluted using the dilution law. 2.5 mL of DPPH solution was added to each crude extract’s solutions of various concentrations; the mixture was kept at room temperature in the dark for 30 min. A control was made by combining 2.5 mL of DPPH solution with 1 mL of 80% methanol. Ultimately, a spectrophotometer set to 517 nm was used to measure the absorbance of the solutions. AA was used as the reference. The extract’s ability to scavenge radicals is indicated by their IC_50_ values.

The extracts′ 50% inhibitory concentrations (IC_50_ values) were determined using a graph that showed percentage inhibition versus concentration. The amount of radical scavenging activity was reported as an inhibition percentage. The concentration of sample needed to scavenge 50% of the DPPH free radical is known as the IC_50_ value. Measurements were taken in triplicate. The IC_50_ of the extracts indicates the corresponding concentration in which the radical scavenging potential is 50%.

The DPPH radical scavenging activity’s percent inhibition (% inhibition) was calculated using the formula below.

(2) % inhibition=Acontrol−AsampleAcontrol×100,

where A_sample_ is the absorbance of the test extract and A_control_ is the absorbance of the control reaction (without the test extract). Based on the graph, the extract concentration that yielded 50% inhibition (IC_50_) was determined. This value was determined using linear regression plots of concentration against the percentage of DPPH scavenged for all test samples [37].

### 2.6. FRAP Assay

The FRAP assay was done according to [1, 38]. Different concentrations of the methanolic and chloroform leaf extracts of *V. sinaiticum* and its several fractions (10–250 μg/mL) were added to 2.5 mL of a 1% potassium ferricyanide [K_3_Fe(CN)_6_] solution and 2.5 mL of a 0.2 M sodium phosphate buffer (pH 6.6). After thoroughly mixing the reaction mixture, it was incubated for 20 min at 50°C. After the mixture had been incubated, 2.5 mL of 10% trichloroacetic acid was added, and it was centrifuged for 10 min at 3000 rpm. A volume of 2.5 mL of distilled water and 0.5 mL of 0.1% ferric chloride were combined with the supernatant (2.5 mL). Using a UV spectrophotometer, the colored solution was measured at 700 nm in relation to the blank using a standard. AA served as the reference. Here, AA was used as a reference standard, reducing the power of the samples, which were comparable with the reference standard.

### 2.7. Antibacterial Activity Test

#### 2.7.1. Preparation of Bacterial Culture

After being cultivated on nutrient agar plates, all of the chosen bacterial strains were given a 24‐h incubation period at 37°C. A portion of these cultures’ colonies were infused into Mueller–Hinton broth and allowed to incubate for a full day at 37°C prior to utilization [39].

#### 2.7.2. Preparation for Test Solutions

To create test solutions (1000 ppm), 10 mg of each crude extract was dissolved in 10 mL of DMSO. The clinical bacterial strains employed for the antibacterial testing activities of the crude extracts were two Gram‐negative bacteria (*Salmonella typhi* and *E. coli*) and two Gram‐positive bacteria (*Streptococcus pneumoniae* and *S. aureus*) microorganisms, which were obtained from the University of Gondar Referral Hospital, Microbiology Laboratory of the Department of Biology. The antibacterial susceptibility test included the standard antibiotic ciprofloxacin as a positive control and DMSO as a negative control [40]. The clinical isolates of *E. coli*, *S. pneumoniae*, *S. aureus,* and *S. typhi* exhibited variable susceptibility profiles, with generally high susceptibility to ciprofloxacin, gentamicin, and meropenem (92%–99%), reduced susceptibility to ceftriaxone (62%–70%), and low susceptibility to ampicillin (55%), particularly among *S. aureus*.

#### 2.7.3. Agar Well Diffusion Method

Using the agar well diffusion method, the antibacterial activity of extracts from *V. sinaiticum* leaves was evaluated against four different strains of bacteria. For the agar well diffusion assay, MHA was equally distributed with 100 μL of each bacterial solution, or 0.5 McFarland’s standard. The wells that the well borer had made were then filled with 100 μL of each crude extract. The positive control in this study was a ciprofloxacin disc, a wide‐spectrum medicine, whereas the negative control was DMSO. For a whole day, the plates were incubated at 37°C. A clear ruler was used to measure the inhibitory zone’s diameter, and the results were expressed in millimeters (mm). For every bacterial strain under evaluation, the tests were conducted three times, and the results were displayed as an arithmetic mean (mean ± SD).

#### 2.7.4. Minimum Inhibitory Concentration (MIC) and Minimum Bactericidal Concentration (MBC)

The antibacterial activity of the methanolic extract of *V. sinaiticum* was evaluated using the agar well diffusion method [41, 42] against four bacterial strains: two Gram‐positive bacteria, *S. aureus* and *S. pneumonia,* and two Gram‐negative bacteria, *E. coli* and *S. typhi*. The bacterial strains were first individually cultured in nutrient broth and then incubated at 37°C for 24 h. Mueller–Hinton agar was prepared and sterilized by autoclaving at 121°C for 30 min, poured into sterile Petri dishes, and allowed to solidify. Subsequently, 100 μL of each bacterial suspension was uniformly swabbed onto the surface of the Mueller–Hinton agar plates. After inoculation, four wells (5 mm in diameter) were aseptically punched into each agar plate using a sterile cork borer.

Aliquots of 100 μL of *V. sinaiticum* plant extracts at concentrations of 100, 50, 25, and 12.5 μg/mL, prepared in dimethylsulfoxide, were added to the wells. DMSO served as the negative control, while ciprofloxacin (5 μg/disk) served as the positive control. The plates underwent a prediffusion of 30 min at 4°C before incubation at 37°C for 24 h. Each extract was tested in triplicate, and the resulting inhibition zone diameters were measured for analysis.

To determine the MBC value, 2 mL samples were plated onto Muller–Hinton agar and incubated at 37°C for 24 h. After incubation, the agar plates were examined to assess the presence or absence of bacteria [43]. The antibacterial activities of *V. sinaiticum* at a concentration of 100 mg/mL were found to be almost comparable to the standard ciprofloxacin (5 μg/mL).

### 2.8. Statistical Techniques

The data on TPCs, TFCs, and antibacterial activities of *V. sinaiticum* leaf extracts were reported as the mean ± standard deviations from three measurements (triplicates). Linear equation of calibration curves and regression coefficients (*R* ^2^) were analyzed using GraphPad Prism software.

## 3. Result and Discussion

### 3.1. Determination of Percentage Yield of Crude Extracts

When isolating phytochemicals from green tissue, the success of the extraction with alcohol is directly related to the extent to which chlorophyll is removed into the solvent, and when the tissue debris, on repeated extraction, is completely free of green color, it can be assumed that all the low molecular weight compounds have been extracted. Terpenoid lactones are obtained from barks by extraction with chloroform. Tannins and terpenoids are treated with less polar solvents [44]. Thus, chloroform and methanol extracts yield (Table 1) were obtained when 200 g of powder plant leaf sample was extracted using a sequential maceration procedure with chloroform and methanol in increasing order of polarity.

**Table 1:** Percent yield of plant extract.

Mass of sample (in grams)	Solvent used	Mass of crude (in gram)	Percent yield of extract (w/w)
200 g leaves of *V. sinaiticum*	Chloroform	2.791	1.395
	Methanol	32.735	16.367

As shown in Table 1, the obtained methanol crude extract has the highest concentration when compared to chloroform, which shows that the extract contains more polar chemicals than nonpolar compounds.

### 3.2. Phytochemical Screening Test

It is crucial to investigate the phytochemical constituents present in the plant species to encourage the appropriate use of phytochemicals and ascertain their potential as the source of novel medications. The qualitative preliminary phytochemical analyses were carried out for chloroform and methanol leaf extracts of the *V. sinaiticum* using the standard procedure [39]. The dominant phytoconstituents of *V. sinaiticum* include phenolic compounds, flavonoids, tannins, saponins, and terpenoids. These compounds are responsible for the plant’s therapeutic properties and are found in the leaves, roots, and other parts of the plant. Methanolic extract of *V. sinaiticum* leaves was found to consist of phytochemicals like phenols, steroids, flavonoids, alkaloids, tannins, and saponins. Nevertheless, flavonoids, saponins, phenols, alkaloids, and tannins were absent from the chloroform solvent extract (Table 2).

**Table 2:** A phytochemical screening test from the chloroform and methanol leaf extract of *V*. *sinaiticum*.

Phytochemical constituents	Test performed	Fraction analyzed		Color observed
		Chloroform leaf extract	Methanol leaf extract	
Flavonoids	Shinoda test	−	+	Pink
Steroids	Salkowski’s test	+	+	Gray colour
Saponins	Froth	−	+	Foam
Phenol	Ferric chloride test	−	+	Dark green
Terpenoids	Salkowski’s test	+	+	Reddish brown
Alkaloids	Wagner’s test	−	+	Reddish brown
Tannins	Lead acetate	−	+	White precipitate

*Note:* + indicates the presence of secondary metabolites. −; indicates the absence of secondary metabolites.

### 3.3. Antibacterial Activity Test

The methanolic extract of *V. sinaiticum* leaf is also reported to possess broad‐spectrum antibacterial activity [45]. The agar well diffusion method was used to assess the antibacterial activity of chloroform and methanol leaf extracts of *V. sinaiticum*. Two Gram‐negative (*E. coli* and *S. typhi*) and two Gram‐positive (*S. aureus* and *S. pneumoniae*) were employed for the determination. The inhibition zone values were measured in millimeters using the plastic ruler. The antibacterial inhibitory values of the chloroform and methanol extracts against the tested bacteria were presented in Table 3.

**Table 3:** Antibacterial inhibitory values (mean + standard deviation, in mm) against four bacterial strains of chloroform and methanol leaf extracts of *V. sinaiticum* and standard ciprofloxacin.

Concentration (μg/mL)	Zone of inhibition (mm) Bacterial strains			
	*S. aureus*	*S. pneumonia*	*Escherichia coli*	*S. typhi*
100	19.3 ± 0.6	17.7 ± 0.6	21.7 ± 0.6	19.7 ± 0.6
50	17.8 ± 0.3	15.5 ± 0.6	21.7 ± 0.6	16.7 ± 0.6
25	11.8 ± 0.3	15.5 ± 0.6	18.3 ± 0.6	11.7 ± 0.6
12.5	10.2 ± 1.3	10.3 ± 0.58	15.7 ± 0.58	10.3 ± 0.6
Ciprofloxacin	20.7 ± 0.1	22.5 ± 0.6	23.6 ± 0.4	28.6 ± 0.6

*Note:* Numbers indicate the mean diameters of inhibition of triplicate experiments ± SD.

Plant extracts are more effective against Gram‐positive bacteria than Gram‐negative bacteria due to differences in their cell envelope structures. Gram‐positive bacteria have a thick peptidoglycan layer that easily absorbs antimicrobial compounds, while Gram‐negative bacteria possess a double‐membrane system with an outer membrane that serves as a barrier, limiting the entry of such compounds [45]. Studies confirm these findings, indicating that lipopolysaccharides in Gram‐negative bacteria inhibit permeability to hydrophobic molecules. Furthermore, phenolic compounds from plant extracts can disrupt membrane integrity and inactivate cellular enzymes, ultimately leading to cell death [46]. These results were consistent with previous studies [13, 39], possibly because of differences in cell structures between these bacteria. The in vitro antibacterial activity of *V. sinaiticum* root diethyl ether, chloroform, acetone, and ethanol extracts at the concentrations of 50, 100, 250, and 500 μg/mL and standard antibiotic ciprofloxacin (20 μg/mL) ranging from 7 mm to 11 mm in diameter with significant results was shown by the ethanolic root extract of *V. sinaiticum* against Gram‐negative bacteria *E. coli* [39].

The antibacterial activity of methanolic leaf extracts of *V. sinaiticum*, as shown in Table 3, exhibited a significant zone of inhibition against Gram‐negative bacteria *E. coli* (21.7 mm) at a concentration range between 50 and 100 μg/mL and moderate antibacterial activity against another Gram‐negative bacterium *S. typhi* (16.7–19.7 mm) at a concentration range between 50 and 100 μg/mL and Gram‐positive bacteria *S. aureus* (19.3 mm) at 100 μg/mL, and the remaining tested bacteria exhibited resistance against the methanolic extract.

The antibacterial activity of *V. sinaiticum* (Table 3) at a concentration of 100 μg/mL was found to be almost comparable to the standard ciprofloxacin (5 μg/mL). The antibacterial activity profile of *V. sinaiticum* against the tested strains indicated that *E. coli* was the most susceptible bacterium of all the bacterial test strains (pictures of antibacterial susceptibility and MIC *V. sinaiticum* extracts for standard bacteria are available in supporting information, Figure SM1).

In general, among the tested bacterial strains, the antibacterial activity was more pronounced on the Gram‐negative bacteria (*E. coli and S. typhi)* than Gram‐positive bacteria (*S. aureus* and *S. pneumonia*). Based on the initial antibacterial screening test of *V. sinaiticum*, further studies were done for the determination of MIC because they were found to be active against bacterial strains. The MICs of the extracts are shown in Table 4 (a photograph of the MIC assay after 24 hrs of incubation is available in the supporting information, Figure SM2). The MIC values indicate that the extracts of *V. sinaiticum* are potent against bacteria. The results agreed with the initial antibacterial screening test results. The lowest MIC value observed was 12.5 μg/mL, which was the MIC value of the methanolic extract of *V. sinaiticum* on *S. pneumonia*. On the other hand, the highest MIC value was registered for *E. coli* (the least sensitive bacterial strain) to the crude extract of *V. sinaiticum*, that is, 12.5 μg/mL.

**Table 4:** MIC and MBC of methanol plant extracts on the bacterial strains.

Bacterial strain		Concentration range	MIC (μg/mL)	MBC (μg/mL)
Gram‐positive	*Staphylococcus aureus*	12.5–100 μg/mL	50	> 50
	*Streptococcus pneumonia*		50	> 50
Gram‐negative	*Escherichia coli*		12.5	> 50
	*Salmonella typhi*		50	> 50

In reported previous studies, the ethanolic extracts of *V. sinaiticum* leaves showed strong antimicrobial activity against *Enterococcus faecalis, Proteus mirabilis,* and *Candida albicans* with inhibition zones of 20.0, 18.0, and 20.0 mm, with MICs and MBCs of 4.0 (8.0), 8.0 (16.0), and 8.0 (16.0) μg/mL, respectively. Also, the extracts exhibited moderate activity against the other test microorganisms. The results demonstrate that the ethanol extract of the aerial parts of *V. sinaiticum* has significant activity and suggests that it may be useful in the treatment of infections [47]. The determined IC_50_ values for AA were 12 μg/mL and plant extract 143 μg/mL [12]. Our findings are like other authors who have reported no significant changes in MIC values of leaf extract.

Within the *Verbascum* genus, the biological activities of *V. sinaiticum* are generally consistent with those reported for other species. Many members of the genus are well known for their anti‐inflammatory, antimicrobial, and wound‐healing properties, suggesting a shared phytochemical and pharmacological profile. For instance, species such as *Verbascum thapsus* and *Verbascum phlomoides* have been widely studied for their analgesic and anti‐inflammatory effects, as well as their traditional use in treating respiratory conditions [48]. *Verbascum qulebrium* ethanol extracts demonstrated superior activity against *Bacillus subtilis* and *Saccharomyces pastorianus* compared to some other *Verbascum* species, particularly in inhibiting *S. pastorianus* [49]*.* The antibacterial activity of *Verbascum thapsus* has been found to be comparable or slightly higher than other *Verbascum* species, such as *V. sinaiticum*, whose root extracts generally show moderate antibacterial activity against various bacteria (e.g., inhibition zones of 7–11 mm) [48, 50]. This overlap indicates that *V. sinaiticum* conforms to the core medicinal characteristics of the genus.

When compared with other well‐known Ethiopian medicinal plants, *V. sinaiticum* similarly demonstrates a wider range of biological activities. For example, *Justicia schimperiana* is recognized primarily for its hepatoprotective properties [51], while *Dodonaea angustifolia* and *Rumex nepalensis* are valued for their antioxidant and antimicrobial effects [52]. In contrast, *V. sinaiticum* integrates multiple pharmacological effects, such as wound healing, antiparasitic, and hepatoprotective actions, within a single species. This multifunctionality enhances its importance in traditional medicine, where accessibility to diverse treatments may be limited.

The strong wound‐healing activity of *V. sinaiticum* is particularly noteworthy and aligns with its ethnomedicinal use in treating skin injuries. This effect may be attributed to its antioxidant and anti‐inflammatory properties, which enhance tissue repair and reduce oxidative stress at the wound site [45].

Overall, *V. sinaiticum* not only reflects the general therapeutic profile of the *Verbascum* genus but also stands out as a pharmacologically versatile species among Ethiopian medicinal plants. Its broad spectrum of activity suggests that it could serve as a valuable source for the development of novel therapeutic agents. Future studies should focus on isolating specific bioactive compounds, elucidating mechanisms of action, and conducting clinical evaluations to validate its efficacy and safety in humans.

### 3.4. Quantitative Analysis

#### 3.4.1. Determination of TPC

The obtained TPC and TFC values of the extracts of *V. sinaiticum* are given in Table 5. The methanol extract exhibited the highest values (TPC = 7.81 ± 0.14 mg GAE/g) (as shown in Figure SM3 in supporting information). The high phenolic and flavonoid content is significant, as these compounds are well known for their antioxidant properties. Phenolic compounds, such as flavonoids, tannins, and phenolic acids, are vital for their free radical‐scavenging capabilities and protection against oxidative stress [6].

**Table 5:** Total phenolic (TPC) and total flavonoids (TFC) contents of chloroform and methanol leaf extracts of *V. sinaiticum*.

*V. sinaiticum* extract	Total phenolic content (mg GAE/g)	Total flavonoid content (mg QE/g)
Chloroform	6.95 ± 0.32	12.13 ± 0.301
Methanol	7.81 ± 0.14	14.20 ± 0.986

The TFC of the extracts was determined as milligrams of quercetin equivalent per gram of dried crude extract (mg QE/g) using the obtained equation *Y* = 0.09952 ∗ *x* − 0.9401, *R* ^2^ = 0.9987) (detailed analytical results are available in supporting information Tables SM1, SM2, SM3 and Figures SM4, SM5, SM6). Accordingly, the highest TFC content (14.20 ± 0.986 mg QE/g) was observed in the methanol extract. The richness of phenolic content in the methanol extract aligns with the extracts’ potent antioxidant activities, as demonstrated in subsequent assays. This high concentration of bioactive compounds suggests that the methanol extract might have the strongest potential for medicinal use, particularly in oxidative stress‐related conditions.

The results are displayed in Table 5 and show that the methanol extract had moderate TPC, measuring 7.81 ± 0.14 mg GAE/g of extract, while the chloroform extract had the lowest TPC, measuring 6.95 ± 0.32 mg GAE/g of extract.

### 3.5. Anti‐DPPH Activity

**FIGURE 1:**
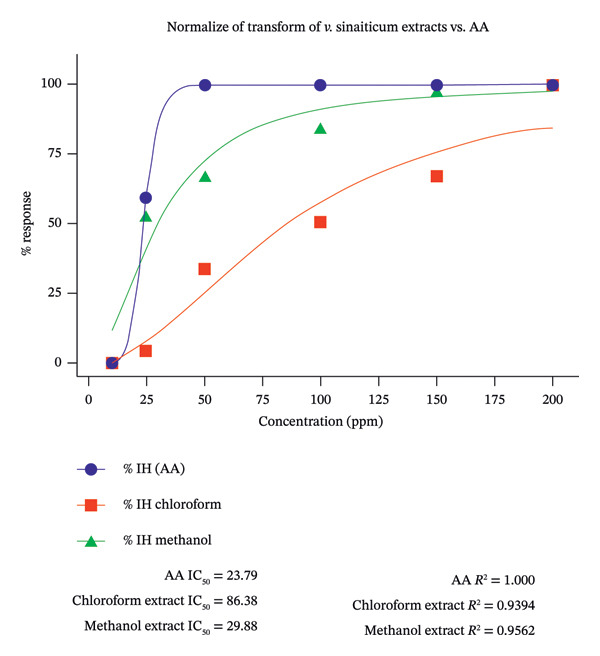
Anti‐DPPH radical scavenging activity percentage versus various concentrations (ppm) of ascorbic acid (AA) and chloroform and methanol leaf extracts of *V. sinaiticum.*

The radical scavenging activity percentage and IC_50_ values against the DPPH radical of the chloroform and methanol leaf extracts of *V. sinaiticum* as well as the standard AA are presented in Table 6 and Figure 1.

**Table 6:** Anti‐DPPH radical scavenging activity percentage versus various concentrations (ppm) of ascorbic acid (AA) and chloroform and methanol leaf extracts of *V. sinaiticum*.

Concentration (AA) ppm	“% radical scavenging” activity of test samples against DPPH radical		
	AA	Chloroform	Methanol
200	97.287	41.26984	58.92707
150	97.112	28.48789	58.08885
100	97.025	22.13868	52.22129
50	96.937	15.53885	44.67728
25	68.503	4.34419	37.9715
10	26.946	2.75689	15.33948
IC_50_	23.79	86.38	29.38

DPPH and FRAP are spectrophotometric assays for assessing antioxidant activity, differing in their mechanisms: DPPH quantifies free radical scavenging (H‐donors) through a colorimetric change (purple to yellow) at approximately 517 nm (a photograph of the FRAP assay is available in supporting information, Figure 7), whereas FRAP evaluates ferric‐to‐ferrous ion reduction (electron donors) by the formation of a blue hue at around 700 nm, indicating distinct antioxidant pathways (hydrogen atom transfer vs. electron transfer). DPPH employs a stable organic radical, whereas FRAP entails metal ion reduction, resulting in variable outcomes, however frequently demonstrating a strong connection for polyphenolic substances. Working solutions of the DPPH and FRAP were used immediately after preparation.

The extract’s ability to scavenge free radicals was assessed using the DPPH test, and the IC_50_ values were used to express the results. As shown in Table 6 and Figure 1 above, the percentage of DPPH radical scavenging activity of standard AA, chloroform, and methanol extracts of *V*. *sinaiticum* leaves was increased as concentrations increased. The plant’s methanol extract had the higher radical scavenging activity with an IC_50_ value of 29.88 μg/mL, while the chloroform extract showed lower activity with a higher IC_50_ value of 86.38 μg/mL. The AA showed the lowest IC_50_ value (23.79 μg/mL). In this investigation the solvent extract shows less antioxidant potential than the reference AA in both situations.

The FRAP assay revealed a dose‐dependent enhancement in the reducing power of the tested sample, indicating increased antioxidant potential with rising concentration, which is consistent with the principle of the FRAP method and previous reports [53]. The capacity of antioxidant compounds to convert the Fe^3+^/ferricyanide complex into its Fe^2+^ (ferrous) form is widely regarded as a key indicator of reducing ability and antioxidant activity [54]. During the FRAP reaction, the initially yellow test solution gradually develops green to blue coloration as the reduction capacity of the extracts or compounds increases. This reduction process, driven by the presence of electron‐donating reductants, leads to the formation of Fe^2+^, which is quantitatively assessed by measuring the intensity of Perl’s Prussian blue complex at 700 nm [55]. Among the tested extracts, the methanolic extract demonstrated the highest antioxidant activity, surpassing the chloroform extract, although both exhibited lower reducing power than the AA standard (Table 7). This observation indicates that polar phytochemicals play a dominant role in contributing to the reducing capacity of the plant extract.

**Table 7:** Concentration and % inhibition and ferric reducing antioxidant power (FRAP) of ascorbic acid, chloroform, and methanol crude extracts.

Concentration (μg/mL)	% reducing antioxidant power		
	AA	Methanol	Chloroform
10	30.5	28.3	21.4
25	32	31.4	27.7
50	52.9	34.6	33.4
100	61.8	60.2	52.9
150	72.4	74.3	60.6
200	86.4	84.2	61.7
250	100	100	100
IC_50_ (μg/mL)	46.2 ± 1.04	80.3 ± 1.40	201.2 ± 1.98
*p* values < 0.05			

**FIGURE 2:**
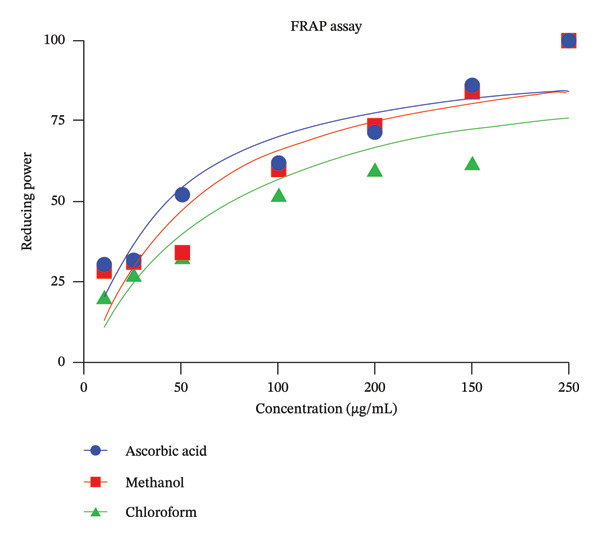
Ferric reducing antioxidant power (FRAP) of ascorbic acids, methanol, and chloroform of plant extracts. Results expressed as the mean ± standard deviation (*n* = 3) at concentrations of 10–250 μg/mL.

The enhanced antioxidant activity of the methanol extract can be attributed to its greater efficiency in extracting polar antioxidant constituents, including phenolic compounds, flavonoids, and reducing sugars, which are known to significantly facilitate ferric ion reduction [18]. In contrast, the chloroform extract showed comparatively weaker FRAP activity, likely due to its limited ability to solubilize polar redox‐active compounds, as chloroform predominantly extracts nonpolar constituents. Consequently, as shown in Figure 2, the overall antioxidant potency followed the order: AA > methanol extract > chloroform extract.

Among the extracts, the methanol extract exhibited the highest antioxidant activity (IC_50_ ≈ 80.3 ± 1.40 μg/mL) compared to the chloroform extract (IC_50_ ≈ 201.2 ± 1.98 μg/mL), although both were less potent than AA (IC_50_ ≈ 46.2 ± 1.04 μg/mL). These findings suggest that polar phytochemicals are the primary contributors to the reducing capacity of the plant extract.

In general, the methanol extract had better DPPH inhibitory activity than the chloroform extract of *V. sinaiticum* leaf, based on the standard level of antioxidant activity proposed [56]. That the smaller the IC_50_ value, the higher the antioxidant activity, where the antioxidant capacity is classified as very strong 50 μg/mL, strong 50–100 μg/mL, moderate 101–150 μg/mL, and weak > 150 μg/mL; and very weak. If the IC_50_ value is > 200 μg/mL. Based on this category, the antioxidant activity of *V. sinaiticum* methanol leaf extract was included in the strong category because the IC_50_ value was in the range of 20–80 μg/L (1 ppm = 1 µg/mL).

The one‐way ANOVA showed a statistically significant difference (*p* < 0.05) among the IC_50_ values of the tested samples, indicating that the antioxidant capacities differed significantly depending on the sample type.

## 4. Conclusion

The *V. sinaiticum* methanol leaf extract showed the highest yield of crude extract (32.73 g, 16.37% w/w) and antibacterial activity as compared to the chloroform extract, which ranged from 24.0 ± 1.0 to 27.0 ± 1.0 against the tested bacteria. The maximum phenolic and flavonoid content was found in the methanol extract (7.81 ± 0.14 mg GAE/g and 14.20 ± 0.986 mg QE/g). The results of the present study showed that the extract of *V. sinaiticum*, which contains the highest number of phenolic compounds, exhibited the greatest antioxidant activity. The high scavenging property of *V. sinaiticum* may be due to hydroxyl groups existing in the phenolic compounds. In general, the plant, *V. sinaiticum* leaf, is rich in phytochemicals, and the presence of these bioactive constituents supports the traditional claim of the medicinal plant. The methanolic extract of *V. sinaiticum* leaf is also reported to possess broad‐spectrum antibacterial activity [45]. This study provides preliminary information for further phytochemical investigation of *V. sinaiticum* to isolate potential antioxidant and antibacterial compounds. Finally controlled human trials are required to confirm the findings from in vitro studies.

## Author Contributions

All the authors made significant contributions to the manuscript and agree to its publication. Birhan Amanie Mekonnen carried out the experimental works and wrote the manuscript. Ayalew Temesgen Wodajo designed the research topic and analyzed the data. Shewaye Lakew Mekuria and Gizachew Mulugeta Manahelohe supervised the experimental works and contributed to reviewing the draft of the manuscript.

## Funding

The author(s) declared that no financial support was received for the research, authorship, and/or publication of this article.

## Consent

The authors have nothing to report.

## Conflicts of Interest

The authors declare no conflicts of interest.

## Supporting Information

Additional supporting information can be found online in the Supporting Information section.

## Supporting information


**Supporting Information** Data used to support the findings of this study are included within the supporting information file. Supporting figures and tables are available in the supporting information and cited in the text.

## Data Availability

The datasets generated and/or analyzed during the current study are included in the manuscript. Moreover, the datasets used to generate figures and results are available from the corresponding author on reasonable request.
