# Supplementary material for: Preliminary Phytoconstituent Analysis and In Vitro Evaluation of Antibacterial and Antioxidant Activities of Verbascum sinaiticum Benth Leaf Extracts
Source: J Trop Med. 2026 Jun 18;2026:6626328. doi: 10.1155/jotm/6626328 (PMC13277388; doi:10.1155/jotm/6626328)
Supplement: Supplementary file 1 — Supporting Information Data used to support the findings of this study are included within the supporting information file. Supporting figures and tables are available in the supporting information and cited in the text. [file JOTM-2026-6626328-s001.docx]

**Supporting Materials:**

**Preparation of standard** :10 mg of Gallic acid standard was added into 100 mL flask, dissolved in 10 mL methanol and distilled water was added up to the mark. To prepare 1000 ppm master stock solution. Then (2.5, 5, 10, 25, 50, 100 and 150 ppm) were diluted using dilution law.

**C_1_.V_1_** = **C_2_.V_2_**

In the formula: **C_1_**: the concentration of the stock standard solution, **V_1_**: the volume to be taken from the stock standard solution, **C_2_**: the concentration of the solution to be prepared, **V_2_**: the volume of the solution to be prepared.

**Preparation of Folin-Ciocalteu Reagent: -** 10 mL of Folin-Ciocalteu reagent is taken into a tape measure, completed to 100 mL volume with pure water and stored in an amber colored bottle.

**Preparation of Na_2_CO_3_ Solution: -** 10 mg of 7.5 % Na_2_CO_3_ weighed and completed to 100 mL with pure water. Dissolution is provided using water bath for 15 minutes

**Preparation of crude extract solution: -**10 mg of each crude extract were dissolved in 10 mL of methanol.

After filling the test tubes with 0.5 mL of the methanolic solution, 3 mL of distilled water and 250 µL of the Folin-Ciocalteu reagent were combined and shaken. 1 mL of 7.5% Na_2_CO_3_ was added after five minutes. It was incubated at room temperature for ninety minutes. The reaction between the phenol and the Folin Ciocalteu reagent produced the vivid blue color. Using a UV- Vis spectrophotometer, the absorbance was measured at 765 nm following incubation. Every experiment was run in triplicate, and the calibration curve was produced using the average results obtained at various gallic acid concentrations. The plant's total phenolic content was measured in milligrams of gallic acid equivalent (GAE) per gram (mgGAE/g) of extract. All the samples were analyzed in triplicate.

**The antibacterial susceptibility test**


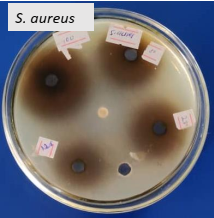

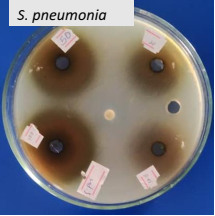

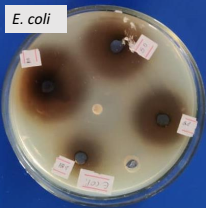

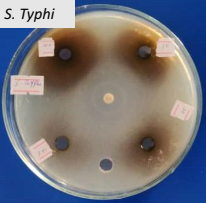


**Figure -SM1**: The antibacterial susceptibility, MIC *V. sinaiticum* extracts for Standard bacteria


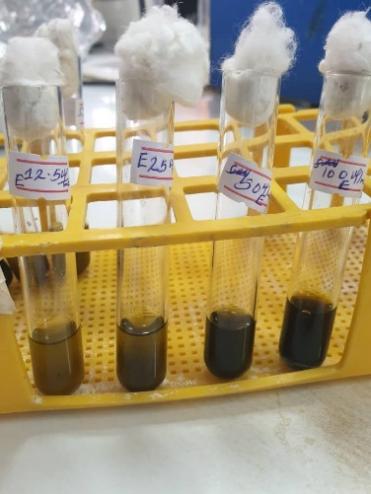


**Figure–SM2:** Photograph of MIC assay after 24 Hrs incubation

**Determination of Total Phenolic Content (TPC)**

The formula **TPC = CV/m** was used to determine the total phenolic contents of all the samples. TPC is equal to the total phenolic mgGAE/g of extract, **C** is the concentration of gallic acid measured in mg/mL from the calibration curve, **V** is the volume of extract measured in milliliters, and **m** is the mass of extract measured in grams^54,55^. The calibration curve is generated on the computer using graph pad prism software; the concentration of the sample is calculated from the obtained calibration equation, as the resulting graph will automatically give the regression equation.

**Regression Equation:** **Y = bx + a** is expressed by the formula. In the formula.

Y: Absorbance; x: Concentration; a: Cutting point and b: Slope of line.

The standard Gallic acid absorbance vs concentration is displayed in Table-SM1.

Table- SM1: Absorbance of gallic acid at different concentrations

| Gallic acid concentration (ppm) | 150 | 100 | 50 | 25 | 10 | 5 | 2.5 |
| --- | --- | --- | --- | --- | --- | --- | --- |
| Absorbance at 765 nm | 0.938 | 0.606 | 0.342 | 0.138 | 0.050 | 0.021 | 0.009 |


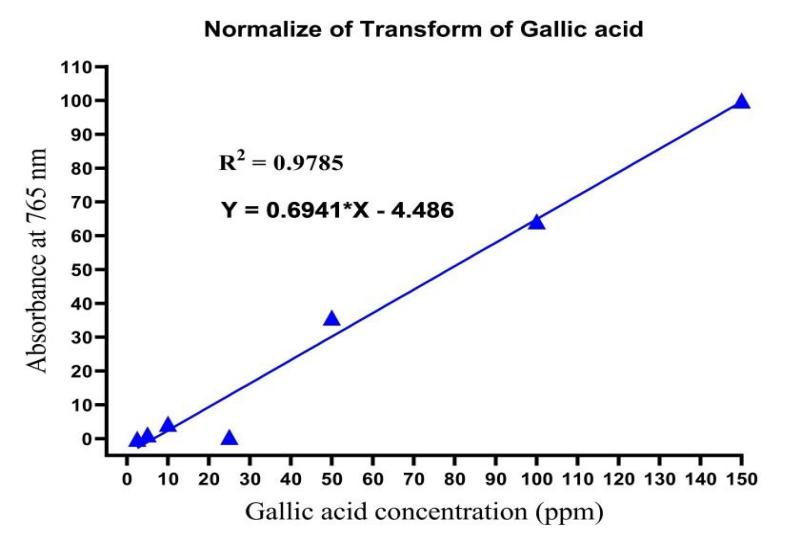


**Figure-SM3:** Normalize of transform of Gallic acid absorbance Vs concentration

As shown in the graph, **“****Y = 0.6941^⁎^x - 4.486”** equation is obtained automatically with Graph Pad Prism software and concentration was calculated from this equation. As the value of R^2^ obtained in the regression calculation approaches to 1, the relationship between absorbance and concentration increases and the accuracy of the result increases.

**Table-SM2**: Absorbance of quercetin at different concentration

| Concentration Quercetin (ppm) | 1000 | 800 | 600 | 400 | 200 | 100 | 80 | 50 | 20 | 10 |
| --- | --- | --- | --- | --- | --- | --- | --- | --- | --- | --- |
| Absorbance at 510 nm | 0.475 | 0.369 | 0.273 | 0.183 | 0.086 | 0.056 | 0.027 | 0.02 | 0.009 | 0.001 |


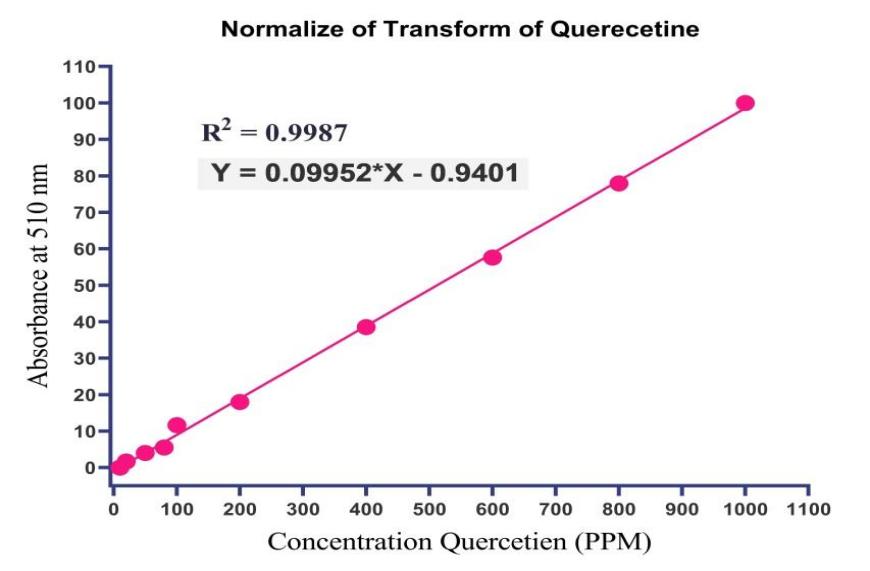


**Figure-SM4:** Normalize of transform of standard quercetin absorbance Vs concentration

Figure SM4 shows the total flavonoid concentration of the crude extract as mg of quercetin equivalents/g of extract. The samples underwent triplicate analysis. The analytical results for the flavonoid concentration of methanol and chloroform aqueous extract are shown in Table SM3. The total flavonoid content of the crude extract is expressed as mg (QE)/g of extract in Figure-SM5 and was determined using the linear regression equation of the standard plot (Y = 0.09952*x-0.9401, R^2^ = 0.9987).

**Table-SM3**: Absorbance of standard ascorbic acid (AA), chloroform and methanol crude extracts of different concentrations at 517 nm

| Concentration (AA) ppm | Absorbance (AA) at 517 nm | Absorbance of chloroform | Absorbance of methanol |
| --- | --- | --- | --- |
| 150 | 0.033 | 0.856 | 0.5 |
| 100 | 0.034 | 0.932 | 0.57 |
| 50 | 0.035 | 1.011 | 0.66 |
| 25 | 0.36 | 1.145 | 0.74 |
| 10 | 0.835 | 1.164 | 1.01 |


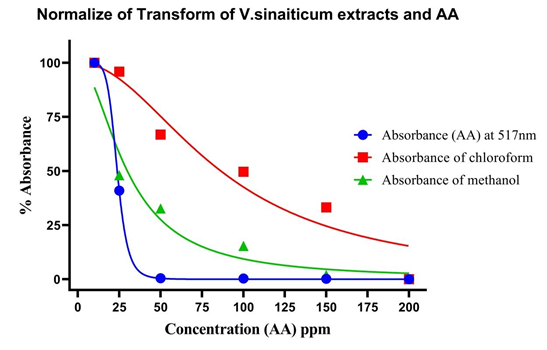


**Figure-SM5**: Normalize of transform of absorbance Vs concentration graph of standard (AA) and plant extracts in DPPH assay


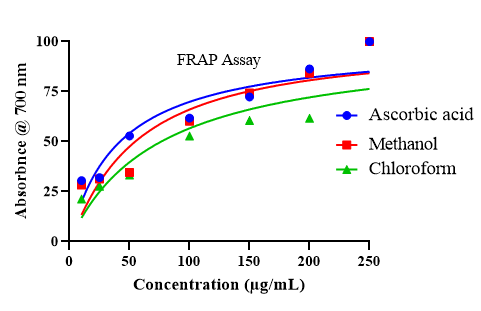


**Figure -SM6**: Ferric reducing antioxidant power (FRAP) of ascorbic acids, methanol, and chloroform of plant extracts. Results expressed as the mean ± standard deviation (n = 3) at concentrations of 10–250 µg/mL.


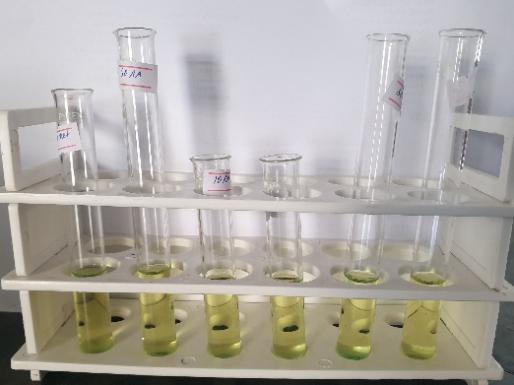

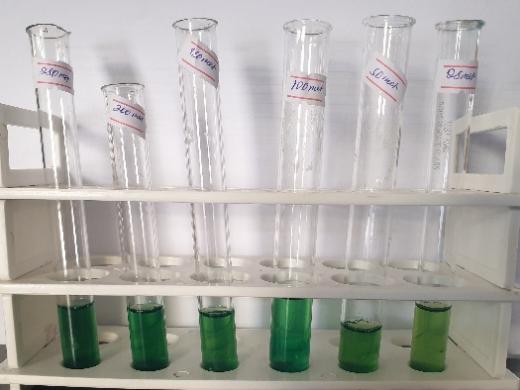


**Figure- SM7:** Photograph of FRAP assay
